# Goldilocks Forgetting in Cross-Situational Learning

**DOI:** 10.3389/fpsyg.2018.01301

**Published:** 2018-08-15

**Authors:** Paul Ibbotson, Diana G. López, Alan J. McKane

**Affiliations:** ^1^Childhood, Youth and Sports Group, Open University, Milton Keynes, United Kingdom; ^2^Theoretical Physics Division, School of Physics and Astronomy, University of Manchester, Manchester, United Kingdom

**Keywords:** cross-situational learning, noise, memory, forgetting, word learning

## Abstract

Given that there is referential uncertainty (noise) when learning words, to what extent can forgetting filter some of that noise out, and be an aid to learning? Using a Cross Situational Learning model we find a U-shaped function of errors indicative of a “Goldilocks” zone of forgetting: an optimum store-loss ratio that is neither too aggressive nor too weak, but just the right amount to produce better learning outcomes. Forgetting acts as a high-pass filter that actively deletes (part of) the referential ambiguity noise, retains intended referents, and effectively amplifies the signal. The model achieves this performance without incorporating any specific cognitive biases of the type proposed in the constraints and principles account, and without any prescribed developmental changes in the underlying learning mechanism. Instead we interpret the model performance as more of a by-product of exposure to input, where the associative strengths in the lexicon grow as a function of linguistic experience in combination with memory limitations. The result adds a mechanistic explanation for the experimental evidence on spaced learning and, more generally, advocates integrating domain-general aspects of cognition, such as memory, into the language acquisition process.

## Introduction

Language learning mechanisms need to be robust enough to acquire normative patterns of use in the face of considerable communicative noise. The term noise is used here to cover a range of learning contexts where the world-to-word relationship is not one-to-one. For example, in principle there are more things in the world that a word could refer to than a speaker intends it to mean (Quine, 1960). This problem of referential indeterminacy, first explored in depth by Wittgenstein (1955), has led some theorists to propose *a priori* constraints that limit the possibilities of referents a learner needs to entertain when acquiring a new word. For example, Markman (1989, 1992) proposed the *whole object constraint* (“assume a novel word refers to the whole object”); the *mutual exclusivity constraint* (“assume novel words refer to unknown objects”) and the taxonomic *constraint* (“labels should be extended to an object of the same kind rather than an object that is thematically related”). Further work relaxed the all-or-nothing requirements of a “constraint” with the more probabilistically applied “principles” (Golinkoff et al., 1994). For example, the *reference principle* (“words map to objects, actions, attributes”) the *extendability principle* (“words extend to other referents”) and the *categorical scope principle* (“words extend to basic-level categories”).

A basic problem with the constraints and principles approach is that for any benefit a bias confers to learn one class of words, it works in the opposite direction for another class. For example, verbs, adjectives, prepositions, and non-typical nouns are quite common in early speech and do not map on to whole objects. Performatives such as *hello, please*, and *thank you*, are again quite common in child directed speech but are not referential. Besides its debatable ability to provide any “in practice” advantage to the learner, the constraints, and principles approach has a more conceptual problem. With every constraint and principle added to the list to explain acquisition, it reduces the power of the theory in predicting findings that are not in the theory itself, and ultimately reduces its falsifiability. What would be more parsimonious and intellectually satisfying are explanations that are simpler, deeper and are independently motivated. One such approach is to see the word learning process as fundamentally integrated with the developing social and cognitive world of the child (Bruner, 1983; Tomasello, 1992, 2003; Nelson, 1996). This predicts that the developing linguistic trajectory of the child should be in part explainable by the developing trajectory of other cognitive faculties such as memory, attention and categorization (Ibbotson and Tomasello, 2009; Ibbotson et al., 2012, 2013a,b, 2018; Kachergis, 2012; Ibbotson and Kearvell-White, 2015; Kachergis and Yu, 2017).

Ever since Ebbinghaus (1913) there has been considerable interest in the role that memory serves in learning. From a developmental perspective, this is particularly relevant as we know infants and children quickly forget information (e.g., Brainerd et al., 1990; Bauer et al., 2000; Rovee-Collier et al., 2001; Vlach and Sandhofer, 2012). In this context forgetting has traditionally—and understandably—been seen as detrimental to learning, reducing the ability to recall known words and to abstract categories. Recently however, the counter-intuitive notion that forgetting is an aid to word learning and concept generalization has received experimental support (forgetting-as-abstraction account; Vlach et al., 2008, 2012; Delaney et al., 2010; Vlach and Sandhofer, 2012; Toppino and Gerbier, 2014; Vlach, 2014). This work suggests spaced learning—distributing learning events over time rather than massing learning together in close succession—allows time for forgetting to occur between learning events. Vlach (2014, p. 165) hints at *why* this regime might improve learning by suggesting “forgetting promotes abstraction by supporting memory for relevant features of a category and deterring memory for irrelevant features of a category.” Here we formally investigate this idea by exploring *how* forgetting could deter memory for irrelevant features when learning a word.

**Figure 1:**
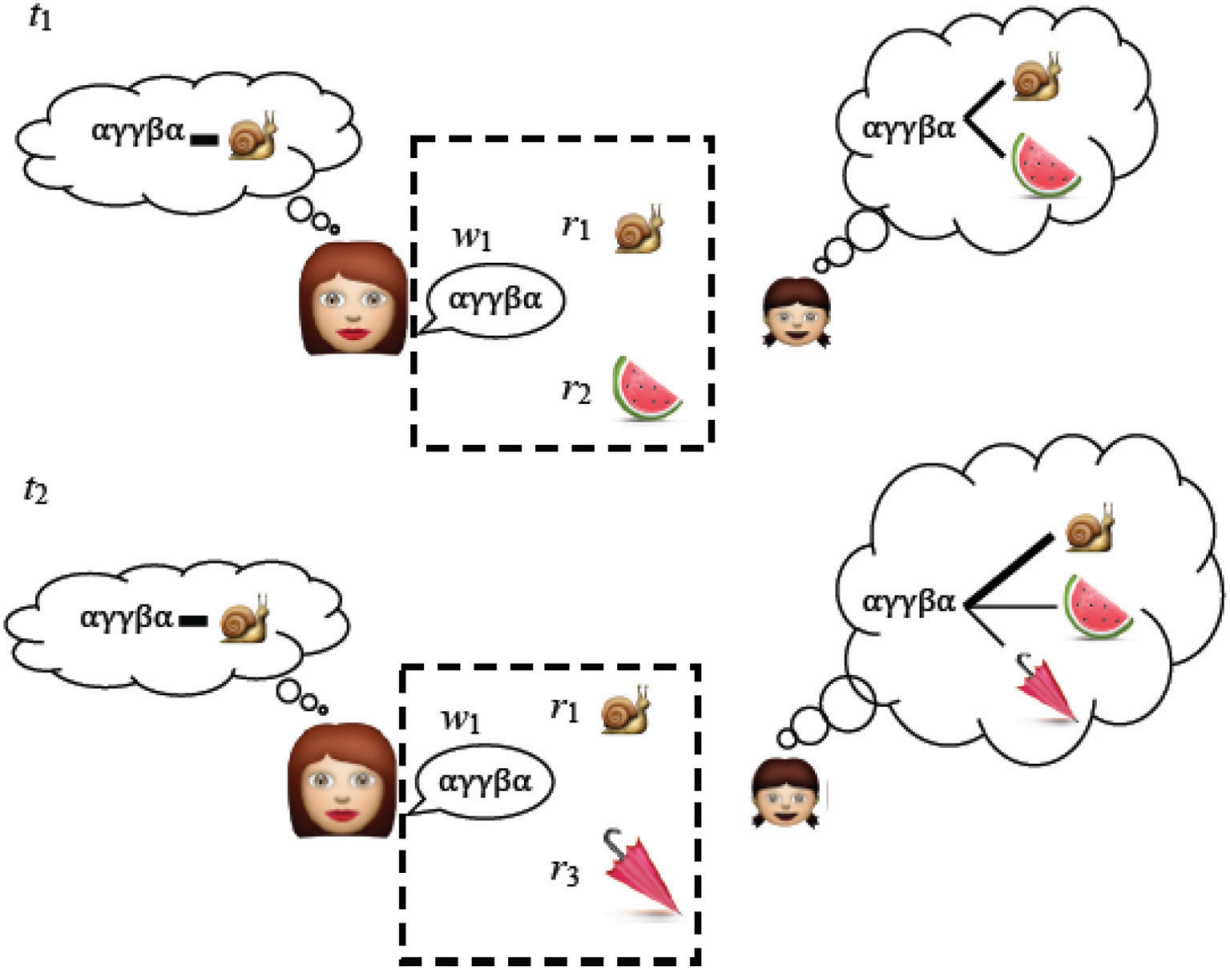
An adult chooses a linguistic expression w_1_ associated with concept r_1_ that they want to communicate. At t_1_ the child does not know whether the novel word w_1_ refers to r_1_ or r_2_ and for now the best she can do is remember the associations between the scene and the words. At t_2_ she hears w_1_ with one object r_1_ familiar from t_1_ and one new object r_3_. XSL works by repeatedly recording the associations between language and the context in which it is used. Over time, the signal (an intentional word-referent pair) is more strongly represented than the noise (an unintentional word-referent pair). Items that appear in the dotted box are the raw data on which the child (or in our case, the system) makes the cross-situational associations.

We investigate this in the context of a cross-situational learning (XSL) model because (a) a large body of evidence suggests that adults, children, and infants are sensitive to the kind of co-occurrence information cross-situational learning capitalizes on, and they use it in word learning (Gleitman, 1990; Pinker, 1994; Siskind, 1996; Akhtar and Montague, 1999; Roy and Pentland, 2002; Frank et al., 2007; Xu and Tenenbaum, 2007; Yu and Smith, 2007; Smith and Yu, 2008; Yu, 2008; Blythe et al., 2010; Cunillera et al., 2010; Fazly et al., 2010; Scott and Fisher, 2012; Vlach and Johnson, 2013; Suanda et al., 2014) and (b) the model gives us a reasonably easy way in which to manipulate forgetting and thus investigate its role on word learning. Informally, cross-situational learning essentially works by using invariant properties in the world-to-word mapping to hone in on the intended meaning, see Figure 1.

Of most relevance to the current study, Tilles and Fontanari (2012) implemented a XSL model that attempted to address the role of memory limitations in the context of word learning. However, forgetting in their model proceeded in a linear fashion. This limits its psychological relevance as ever since Ebbinghaus' (1913) widely replicated findings on forgetting curves showed, forgetting happens in a non-linear fashion, occurring most rapidly right after learning occurs and slowing down over time. Yurovsky and Frank (2015), improved the plausibility of memory decay in a XSL model by formalizing it as a power function (after Murdock, 1982; Anderson and Schooler, 1991; Shiffrin and Steyvers, 1997) but they did not explicitly compare the effect of forgetting vs. no forgetting, as we do here (see also Kachergis et al., 2012). Their focus was on determining whether learners accumulate graded, statistical evidence about multiple referents for each word (e.g., Vouloumanos, 2008; McMurray et al., 2012; Yurovsky et al., 2014) or track only a single candidate referent (e.g., Medina et al., 2011; Trueswell et al., 2013). Interestingly, they found cross-situational learning involves elements of both types, but the success of learning, importantly for this study, depends on limited attention and memory. Here we extend this by implementing two different versions of forgetting in our model, first, a relatively naive model of forgetting and second, a more psychologically plausible model based on an exponential decay function and other aspects of memory performance that were not present in previous studies (Kachergis et al., 2012; Tilles and Fontanari, 2012; Yurovsky and Frank, 2015) (explained in detail in section The Forgetting Mechanisms).

The question that follows from this is: Given that there is referential uncertainty when learning words (noise), to what extent can forgetting filter some of that noise out, and be an aid to learning? In what follows we outline how forgetting interacts with noise conceived of in three different ways: referential ambiguity (“what a speaker intends to refer to vs. what they could be referring to”) within-speaker variance (“the same person referring to the same object in different ways”); between speaker variance (“different people referring to the same object differently”).

## Methods

### The lexicon

We consider the interactions between a child and a community of adult speakers. To start with we assume that all adults are identical in their language use (in section Between-Speaker Variance we shall vary the degree to which adults share a lexicon). The adult lexicon *A*(*r, w*) = *P*(*w*|*r*) is a list of probability distributions over words *w* (*w* = 1, 2, …*W*); one distribution for each referent *r* (*r* = 1, 2, …*R*). That is, *A*(*r, w*) is the probability that the adult will utter the word *w* when talking about referent *r*. This implements our first level of communicative noise, referential ambiguity, because, there are more referents possible than there are words. These distributions are defined once and remain constant throughout the simulations, so they can be considered to be parameters of the model.

For any adult individual their language is indeterminate in the sense that there is not a one-to-one mapping between a word and a referent; this implements our next level of noise, within-speaker variance. In everyday communicative contexts, the same speaker is not guaranteed to use the same word for a given referent. For example, a waitress might refer to a particular customer as *the postman, John, that man, him* or even *the sun glasses* as in *the sunglasses never leaves a tip*. Despite the variation in linguistic form, interlocutors coordinate their representations so that the same referent is identified across multiple situations. An example of an adult lexicon is given in Table 1.

**Table 1:** Example of an adult lexicon *A*(*r,w*).

	**Words**							
**Referents**	**1**	**2**	**3**	**4**	**…**	**8**	**9**	**10**
1	0.75	0.25						
2	0.25	0.5	0.25					
3		0.25	0.5	0.25				
4			0.25	0.5	…			
…				…	…	…		
8					…	0.5	0.25	
9						0.25	0.5	0.25
10							0.25	0.75

*In this case there are 10 referents and 10 words and the matrix is tri-diagonal. Each line corresponds to a referent r. The elements on each line sum up to 1 and they constitute a probability distribution over words: element (i, j) is the probability that word j will be uttered when talking about referent i. Non-specified elements are null. Note that each column is not a probability distribution over referents, though in this simple example columns happen to add up to 1*.

For the time scale that a child acquires her language (and the time scale used in our simulations) we can approximate the group level norms as stable (see Baxter et al., 2006, 2009 for statistical approaches to modeling normative change). This means for any adult individual in our model their language does not evolve over time and by implication nor does the group. The lexicon we implement in this model has a vocabulary of 10 words. The model offered here is not intended to accurately represent all aspects of a child's word learning experience. It is meant to be representative enough to explore how forgetting and speaker variance work in principle, and there are reasons to assume that results from XSL models with small vocabularies are scalable (For example, Blythe et al., 2010 demonstrated mathematically that there is no inherent combinatorial barrier preventing XSL models operating under referential uncertainty from scaling up to full-size lexicons).

We define the child's lexicon *C*(*r, w*)(*t*) in a similar way, so that *C*(*r, w*)(*t*) = *Q*(*w*|*r*)(*t*), but these quantities change in time as the child learns: they constitute the variables of our dynamical system. They represent the associative strength between words and referents in the child's memory at a given time *t* since the beginning of the learning process; the child begins the learning process with no associations between words and referents. In practice element *C*(*r, w*)(*t*) is computed as the (integer) number of tokens *c*(*r, w*) that the child has collected up to time *t* (which is altered by memory loss; see explanation in section The Forgetting Mechanisms below) divided by the number *n*(*r*) of occurrences of referent *r*, *C*(*r, w*) = *c*(*r, w*)/*n*(*r*).

The group lexicon *G*(*r, w*) is the (normalized) sum of the adults' lexicons and represents the norm of the community of speakers. The goal of the learning process is to allow the child to build up a lexicon as close to the group lexicon as possible.

### The learning algorithm

The dynamics of the system take place in the child's lexicon and involves two main processes (1) the acquisition of tokens—via exposure to adult utterances (in the presence of pairs of referents, an intended one and an incidental one)—and (2) the loss of tokens (forgetting). The state of the child's lexicon at a given time is the dynamical result of those two opposing processes. Everything it learns about the adult language, it does so via experience of “usage events,” implemented in this model as presentations of (ambiguous) referents and words. What a word means in this language is the sum total of usage events it appeared in. In terms of the actual simulation procedure, each iteration (child-adult encounter) consists of the following steps:

- Pick a random adult with whom the child will interact (this step only applies where we introduce between-speaker variance; until then it is enough to consider a single adult).
- *draw_refs*: Draw two random referents, *R*1 and *R*2, from a uniform distribution (other shapes will be explored in section Between-Speaker Variance), without replacement.
- *draw_word*: Draw a word *W* from the line in the adult's lexicon that corresponds to referent *R*1, i.e., from the distribution *A*(*R*1, *w*) (see Table 1 for an example of an adult lexicon). This is the word uttered by the adult in the presence of both referents *R*1 (the “target” referent, that the speaker intends to refer to) and *R*2 (the “distractor” referent that gives rise to a spurious association; see Figure 1).
- *record_tokens*: The child associates the two observed referents to the word she has heard: add 1 to the quantity *c*(*R*1, *W*) and to the quantity *c*(*R*2, *W*).
- *give_mark*: Measure how much the child has learnt (see section Measures below).
- *Apply naïve_forget OR Ebbinghaus_forget regime*: See details in section The Forgetting Mechanisms below.

The steps above describe how referential ambiguity is implemented in our model. Following Blythe et al. (2010) we do not assume any relationship between the sets of incidental meanings associated with different target words, that is the distractor or “noisy” word-referent associations. There may be complete overlap between some sets of incidental meanings or no overlap at all, because the distractor referent *R*2 is picked at random each time.

### The forgetting mechanisms

We consider in turn two alternative forgetting mechanisms, which we shall refer to as “naive forgetting” and “Ebbinghaus forgetting” respectively.

The “naive forgetting mechanism” consists in deterministically removing one token from every non-zero entry in the child's lexicon *c*(*r, w*) every *m* iterations, where *m* is the so-called memory parameter. This mechanism is naive in the sense that the number of tokens *c*(*r, w*) decreases linearly in time (in the absence of new evidence), at a rate (1/*m*) which is independent of the current number of tokens, as well as being independent of the rate at which tokens are added to that element of the child's lexicon. We use this mechanism as a baseline against which to compare the following more complex, non-linear forgetting mechanism and to replicate the memory implementation of Tilles and Fontanari (2012).

The “Ebbinghaus forgetting mechanism” is inspired by the findings of Ebbinghaus (1913), and subsequent researchers who formalize human memory performance as a non-linear function (Murdock, 1982; Anderson and Schooler, 1991; Shiffrin and Steyvers, 1997; Yurovsky and Frank, 2015). For example, one model which has had success in capturing the effects of practice and the effects of retention interval—namely that repetition improves recall, and increased temporal spacing improves recall—is the ACT-R model (e.g., Anderson and Lebiere, 1998). ACT-R's activation equation represents the strength of a memory item as the sum of a number of individual memory strengthenings, each corresponding to a past practice event. Using a modified ACT-R model Pavlik and Anderson (2005) accounted for standard spacing effects in various conditions and showed that wide spacing of practice provides increasing benefits as practice accumulates. They extend ACT-R's activation equation by introducing a variable decay-rate function. According to this mechanism, the forgetting rate for each presentation of a memory chunk is a function of the activation of the chunk at time of presentation. We implement a similar trajectory of forgetting in our discrete-token framework by letting the forgetting of tokens happen stochastically at a constant rate *per token* per timestep, so that the time at which the token will disappear follows a decreasing exponential distribution. So we assign to each word-referent token a small probability *d* per timestep that it will be deleted from the child's memory (weakening the associative strength of that word-referent pair). The probabilities per timestep to increase or decrease the number of tokens *c*(*r, w*) by one unit can be written $p_{r,w}^{+1}=$ *p*_*r, w*_ and $p_{r,w}^{-1}=d_{r,w}\cdot c\left(r,w\right)$ respectively. Note that *p*_*r, w*_ also depends on the particular word-use distribution under consideration (see section Between-Speaker Variance and Appendix 1 in Supplementary Material).

Ebbinghaus also demonstrated that it takes longer to forget material after each subsequent re-learning. In our model, unlike Yurovsky and Frank (2015) and Tilles and Fontanari (2012), we therefore keep track of the total number of tokens that have ever been added to that referent-word slot, *N*_*r, w*_(*t*) (“repetitions”). The probability of forgetting each token, *d*_*r, w*_, decreases as *N*_*r, w*_ grows. Also, the forgetting rate should depend not on the absolute number of times that a given word-referent association has been heard, but on its *relative* frequency with respect to the total number of tokens heard so far, $N\left(t\right)=\underset{r,w}{\sum }N_{r,w}\left(t\right)$. Care must be taken in order to avoid introducing an undesired time-dependence. For this reason, we chose an exponential decrease of the forgetting rate with the relative number of repetitions, as follows:

(1) $$d_{r,w}\left(N_{r,w}/N\right)=\;\frac{1}{d_{0}}\exp\left(\frac{-N_{r,w}/N}{d_{1}}\right).$$

**Figure 2:**
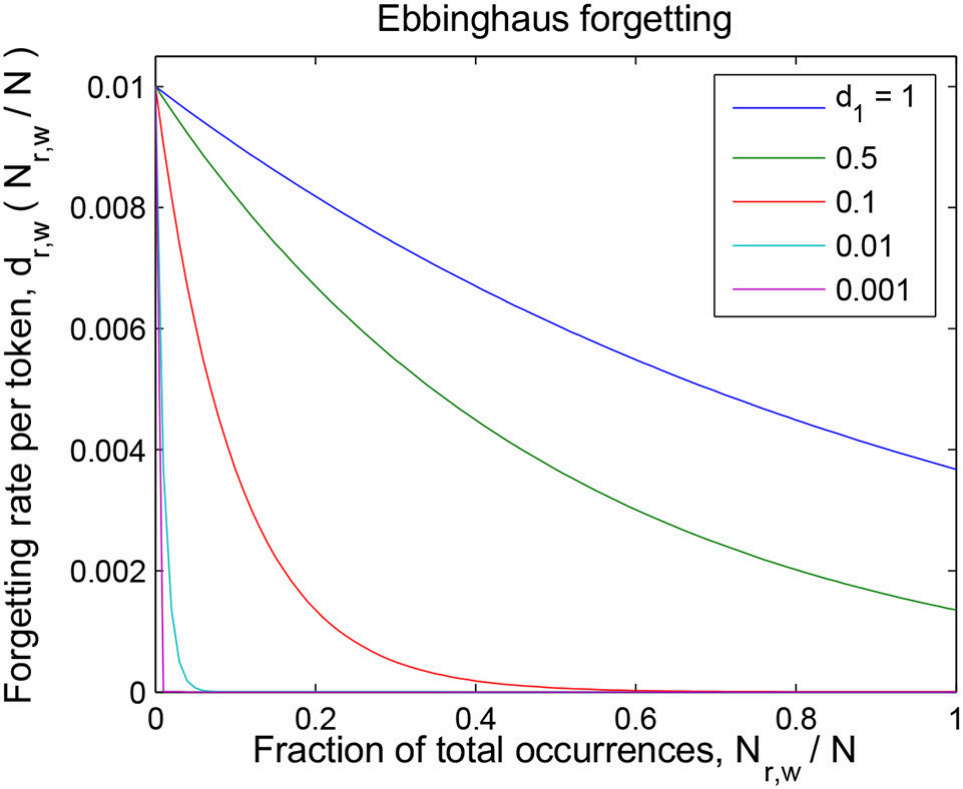
Details of the Ebbinghaus forgetting mechanism described in section The Forgetting Mechanisms. The forgetting rate of a token is 1/*d*_0_ (= 0.01 in this graph) the first time it is encountered, and then decreases with the relative number of repetitions of that word-referent pair. The scale of this decrease is given by parameter *d*_1_ (see Equation 1).

Parameter 1/*d*_0_ is the forgetting rate of seldom-encountered word-referent associations (and therefore it is the forgetting rate of a word-referent token which has just been encountered for the first time). Parameter *d*_1_ governs the drop in the forgetting rate of often-encountered tokens with respect to that of seldom-encountered tokens; when the evidence for a word-referent association accounts for a fraction *d*_1_ of the total evidence collected by the child, the forgetting rate for those tokens will be reduced to 36% (*e*^−1^) of the initial rate 1/*d*_0_. See Figure 2 for an example of such a curve with fixed initial forgetting rate 1/*d*_0_ and for different values of the relative-repetition-scale *d*_1_.

### Measures

We consider the dynamic child lexicon *C*(*r, w*)(*t*) to be an approximation of the “true” static group lexicon *G*(*r, w*). In order to evaluate how good that approximation is, i.e., how much the child has learnt by a given time *t*, we employ the following three complementary measures (for ease of writing we omit their time dependence).

1. Child errors. For each referent *r*, we define an error committed by the child as
  (2) $$\begin{array}{l} E\left(r\right)=\;\frac{1}{W_{0}}\;\overset{W_{0}}{\underset{w_{0}=1}{\sum }}C\left(r,w_{0}\right)\;, \end{array}$$
  where the sum is over the words that should never be used to refer to *r*, according to the group lexicon, {*w*_0_} = {*w* | *G*(*r, w*) = 0}. Then averaging over referents, $E=\;\frac{1}{R}\;\overset{R}{\underset{r=1}{\sum }}E\left(r\right).$ The output of this measure is interpreted as a probability of the child forming an error, defined as an association between a word and a referent that is not present in the adults' lexicon (a zero entry in Table 1).
  We assume that there is more to learning than the absence of errors so the next two measures give us different perspectives on the type of relationship between the child's lexicon and the adults'; one from the perspective of significance difference (Chi-squared) and one from the perspective of strength of association (Pearson's correlation coefficient).
2. Chi-squared. For each referent *r*,
  (3) $$\chi^{2}\left(r\right)=\;\sum_{w=1}^{W}\frac{{\left[C\left(r,w\right)-G\left(r,w\right)\right]}^{2}}{G\left(r,w\right)},$$
  Then averaging, $\chi^{2}=\;\frac{1}{R}\;\overset{R}{\underset{r=1}{\sum }}\chi^{2}\left(r\right)$. Chi-Squared essentially tests whether the distributions which constitute the child's lexicon are significantly different from those of the adults. The output of this measure varies between 0 (tending toward a non-significance difference between adult and child lexicons) to 1 (tending toward a significant difference).
3. Pearson's correlation coefficient. For each referent *r*,
  (4) $$\begin{array}{c} P(r)=\;\frac{\sum_{w=1}^{w}(C(r,w)-\langle C_{r}\rangle )\;(G(r,w)-\langle G_{r}\rangle )\;}{\sqrt{{\sum_{w=1}^{W}\left(C(r,w)-\langle C_{r}\rangle \;\right)}^{2}}\;\;\sqrt{\sum_{w=1}^{W}{\left(G(r,w)-\langle G_{r}\rangle \;\right)}^{2}}} \end{array}$$
  where the average of *C* is given by
  $$\langle C_{r}\rangle =\;\frac{1}{W}\sum_{w=1}^{W}C(r,w).$$
  The coefficient is then averaged over referents, $P=\;\frac{1}{R}\;\overset{R}{\underset{r=1}{\sum }}P\left(r\right)\;.$ Pearson's correlation coefficient tests the strength of association between the child's and adults' lexicon. The output of this measure varies between −1 (perfect negative linear relationship) to 0 (no linear relationship) to +1 (perfect positive linear relationship).

Together these three measures show us to what extent the child's lexicon has converged on that of the adults'.

## Results and discussion

### The role of forgetting

First we report the learning curves of the XSL model for both of the naive and Ebbinghaus forgetting mechanisms. The learning curves are plots of performance measures of the child (Equations 2–4) as a function of time *t*, where *time* stands for the number of iterations of the simulation algorithm of section The learning Algorithm. Less formally, *time* represents the number of adult-child “interactions.” Because each interaction implies the exposure of a word to the child, *time* here is identical to *corpus size*, defined as the number of (not necessarily unique) words that the child has been exposed to.

**Figure 3:**
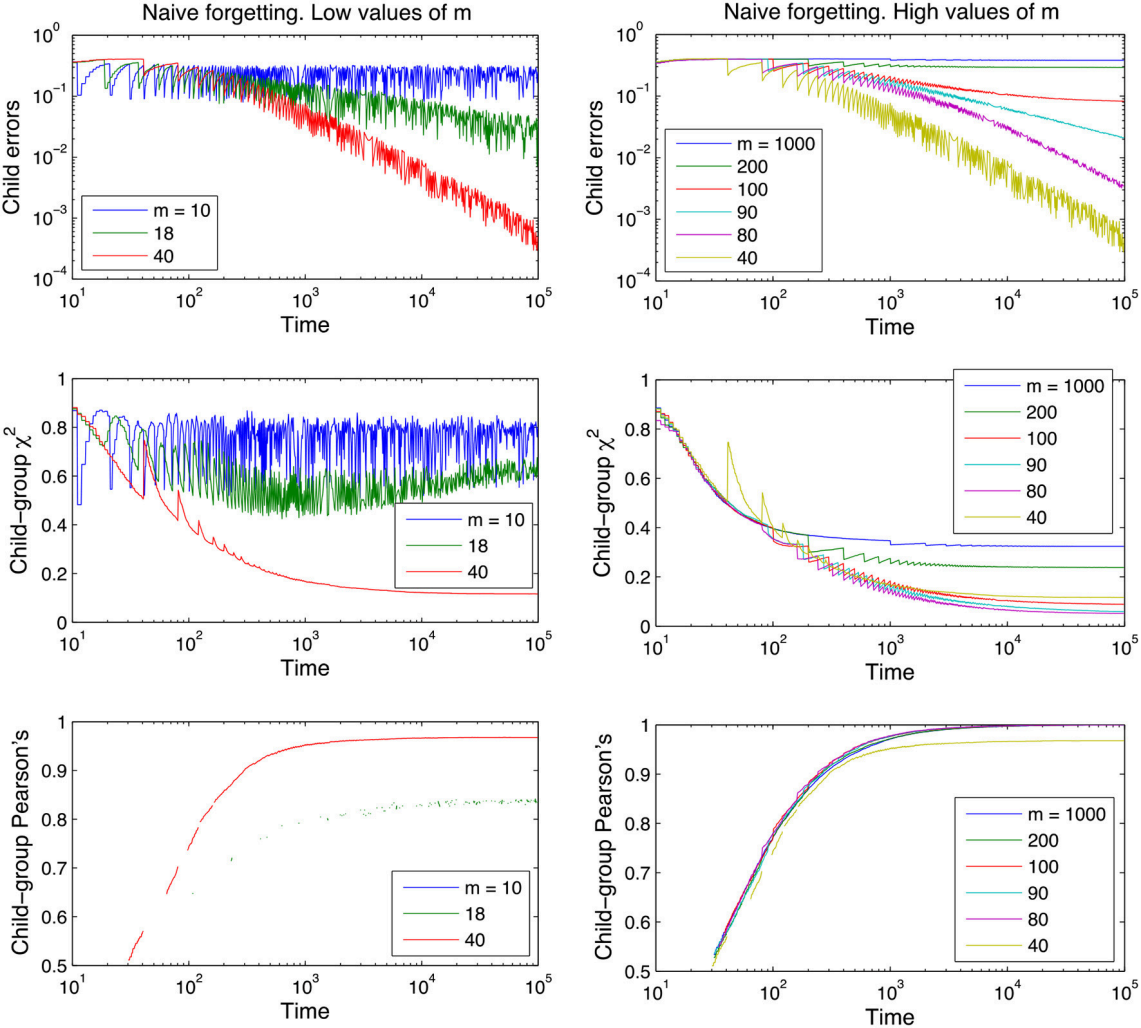
Learning curves for the naive model of forgetting, for different values of the memory parameter *m*. Performance measures are plotted on the y-axis, time is plotted on the x-axis on a logarithmic scale. For ease of inspection we separate the graphs into low values of *m* **(Left)** and high values of *m* **(Right)**. The curves shown are averages over 100 runs for each parameter value; the lexicon used is tri-diagonal as in Table 1 and contains 10 words, and no between-adult diversity is considered.

**Figure 4:**
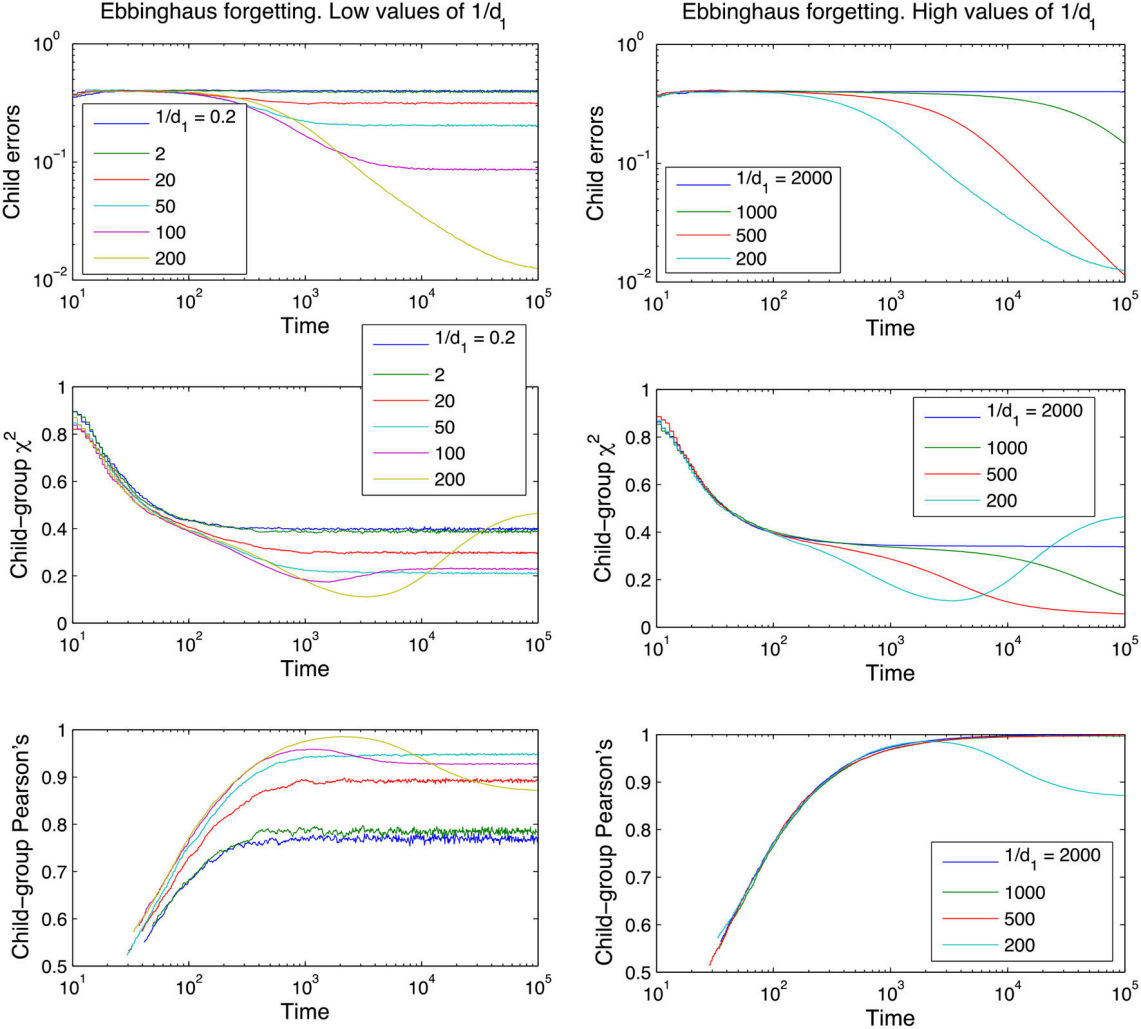
Learning curves for the Ebbinghaus model of forgetting, for different values of the parameter 1/*d*_1_ and using *d*_0_ = 0.01 (see Equation 1 in section The Forgetting Mechanisms). Performance measures are plotted on the y-axis, time is plotted on the x-axis. Curves shown are averages over 100 runs, using a 10-word tri-diagonal lexicon and no between-adult diversity.

Figures 3, 4 show that the model learns incrementally to approximate the adult lexicon as demonstrated by the reduction in child errors to negligible amounts (10^−2^) and the relationship between the child lexicon and the adult lexicon becoming stronger over time (cf. section Measures). By using the logic of XSL the child lexicon is approximating that of the adult lexicon and in this regard we have shown that our model is robust to the effects of referential ambiguity and within-speaker variance implemented in the learning procedure. Like Siskind (1996), Yu (2008), and Fazly et al. (2010) we have also shown that the learning rate increases quickly early on in development and then gradually stabilizes. This is important for two reasons. First, it shows that despite the constant revision of lexical knowledge inherent in the cross-situational mechanism, this does not undermine the consolidation and stabilization of lexical learning (c.f. the problem of catastrophic inference observed in many connectionist models). Second, the general shape of the developmental trajectory is similar to that of the developmental data from longitudinal studies of vocabulary acquisition, where growth is slow in the beginning, accelerates, and then levels off again (e.g., Kamhi, 1986; Gopnik and Meltzoff, 1987; Reznick and Goldfield, 1992). Importantly, our model replicates this trajectory yet has no need for the cognitive constraints or biases that have been proposed to account for this “vocabulary spurt,” for example; a shift from associationist to a referential word meaning mechanism (Nazzi and Bertoncini, 2003); a realization that objects have names (Kamhi, 1986; Reznick and Goldfield, 1992); the development of categorization abilities (Gopnik and Meltzoff, 1987); or the onset of word learning constraints (Behrend, 1990). Following Huttenlocher et al. (1991) and Fazly et al. (2010) we interpret this developmental trajectory as more of a by-product of exposure to input, where the associative strengths in the lexicon grow as a function of linguistic experience. This interpretation seems to fit with the design of our model where learning takes place without incorporating any specific cognitive biases or constraints and without any prescribed developmental changes in the underlying learning mechanism.

**Figure 5:**
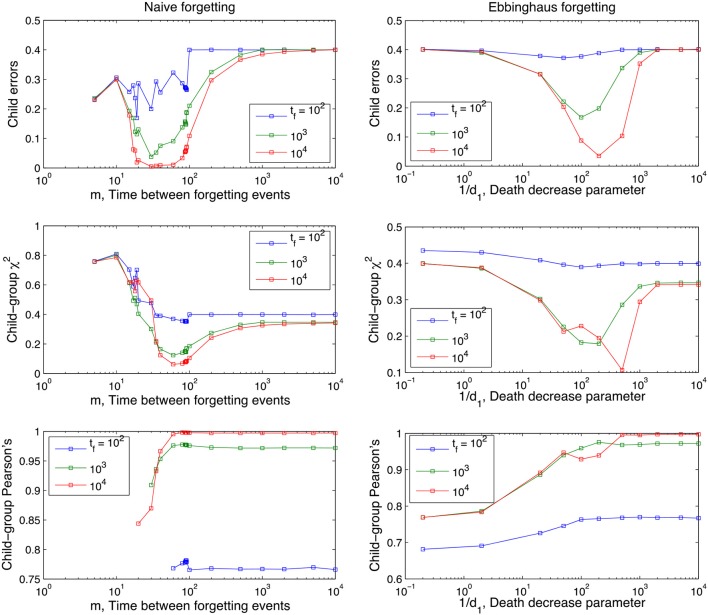
Performance of the Naive and Ebbinghaus forgetting models as a function of the memory parameters (*m* and 1/*d*_1_ respectively) and developmental time points *t*_*f*_ at which the child lexicon is evaluated with respect to the group one. Averages over 1,000 runs, rest of parameters as in Figures 3, 4.

Figures 3, 4 also clearly demonstrate that cross-situational learning is dependent on the balance between forgetting and remembering or the storage-loss ratio. On first inspection there appears to be a critical window between extremes of the memory parameters (high vs. low in Figures 3, 4) where learning is optimal. To confirm whether this is true (and for which developmental time periods) we plotted (Figure 5) cross-sections of the learning curves displayed in Figures 3, 4, to gain a more subtle division of forgetting than “high” vs. “low.” This allows us to see more clearly how the parameters affect learning for a given moment in time.

Figure 5 shows that the long-term dynamics of the child errors in the naive forgetting model seem to fall into three different regimes, depending on the value of the memory parameter *m*:

$$E\left(t\to \infty \right)\;\sim \;\{\begin{array}{ll} constant\;\left(m\right)>0, & m<M_{0}\\ t^{-b}\to 0, & M_{0}<m<M_{1}\\ constant^{\prime }\left(m\right)>0, & m>M_{1}. \end{array}$$

By inspection, *M*_0_ ≈ 17−19 and *M*_1_ ≈ 80−90 for the parameter values used (number of words in lexicon, number of referents shown at once, etc).

If forgetting events are too frequent (low values of *m*), the lexicon never accumulates enough experience (word-referent pairs) to approximate the adult lexicon. In other words, there is not enough time for the cross-situational learning mechanism to build up a strong enough signal before the signal is deleted. Perhaps less obviously, if forgetting events are too rare (high values of *m*), the errors that are learnt (spurious word-referent associations) persist as frozen background noise in the child's memory alongside the correct word-referent mappings dampening the overall performance of the model. These different regimes are further illustrated in Appendix 2 (Supplementary Material).

Presumably the limiting value of the child errors when there is no forgetting at all (*m* → ∞) would be higher the larger the number of referents that were visualized simultaneously (e.g., two instead of one distractor, keeping all other parameters equal); this is essentially what Fazly et al. (2010) found, namely cleaner input (less distractor items) made word learning easier. The limiting value of the child errors would also be higher if the language itself were more complex, in the sense that the adult lexicon distributions *A*(*r, w*) were wider or even had several peaks.

In summary, the U-shaped function of the Child Errors in Figure 5 points to a “Goldilocks” zone of forgetting: an optimum store-loss ratio that is neither too aggressive or too weak, but just the right amount to produce better learning outcomes. Fundamentally, the advantage of a certain degree of forgetting over no forgetting at all is due to the interaction between the XSL mechanism and the noise-to-signal ratio. Across different situations the noise levels are lower than the signal levels (intended word-referent pair) because we assume people use labels across situations with some consistency (but not entirely consistently either). Under these assumptions, forgetting disproportionally affects the noise as it has fewer tokens to delete from experience than the signal and will therefore more frequently approach a zero association. In the case of spurious associations this effect directly improves the performance of the model as any non-zero associations that exist in the child that do not exist in the adult are recoded as errors. In other words, forgetting acts as a high-pass filter that actively deletes (part of) the referential ambiguity noise which masks the adult lexicon as seen from the perspective of the child.

It is noticeable from Figure 5 that there is a slight shift in this optimum storage-loss ratio for learning from more aggressive forgetting early on in development to less aggressive later on in development. In other words, it pays to forget more aggressively early on in language development and this facet of the model fits with an improving trajectory of memory performance as the child develops (Brainerd et al., 1990; Bauer et al., 2000; Rovee-Collier et al., 2001; Vlach and Sandhofer, 2012). It also serves to underscore the complex dynamics of the component parts involved in the process of word learning a memory: what is an optimum store/loss ratio at one point in development is not necessarily true across development.

**Figure 6:**
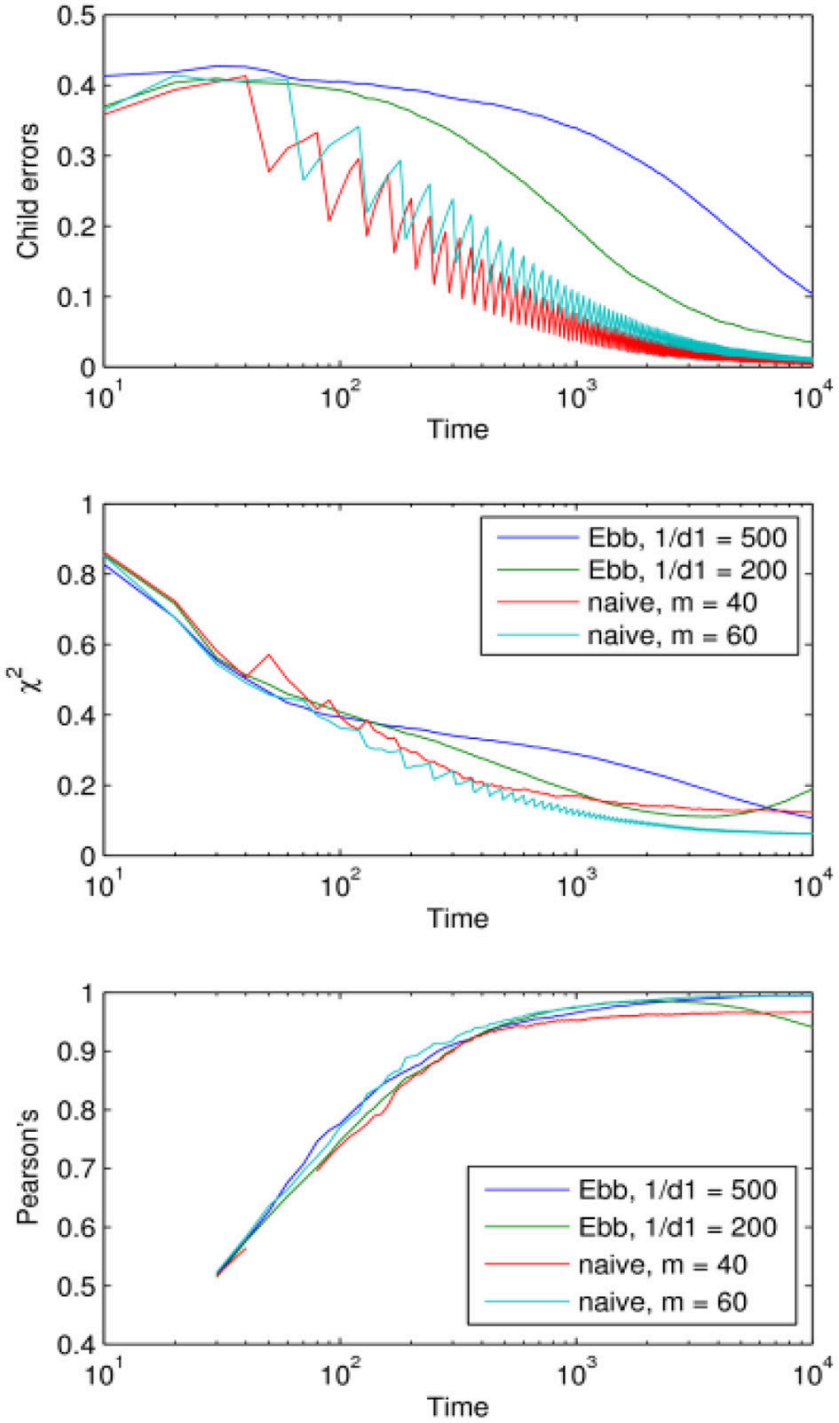
Comparison of the performance of the naive and Ebbinghaus forgetting mechanisms: learning curves corresponding to the optimal parameter values (optimal within the region explored in Figures 3, 4).

Finally we present a direct comparison between the naive and the Ebbinghaus models of forgetting by choosing the optimum memory parameters based on how the models performed after 10^4^ iterations—the stopping point in this simulation (Figure 6).

One might have expected the Ebbinghaus model to have performed slightly better due to the rapid decay of associative strength it applies to infrequently encountered items, thus dampening the effects of noise. Figure 6 shows no overall advantage for the Ebbinghaus model using these optima for the time scale of 10^1^-10^4^ iterations. It appears the added complexity of the Ebbinghaus mechanism does not translate into a significant improvement in performance when compared with the naive mechanism. One potential reason for this is that the “naive” mechanism actually incorporates one aspect of the more sophisticated exponential decay function model. Its forgetting rate is 1/*m* *per element* in the child's lexicon matrix, which translates into a *per-token* forgetting rate that decreases as the number of tokens *c*(*r, w*) grows. So the naive mechanism actually shares this reinforcement characteristic of the Ebbinghaus mechanism yet is much simpler.

Importantly for the main point we are establishing here both models learn the adult lexicon in the face of communicative noise and both show an optimum storage-loss ratio for cross-situational learning. We demonstrate here for a XSL model what Elman (1993) showed for a connectionist model of grammar learning; implementing a more plausible (and limited) memory capacity into a model can actually have pay-offs in terms of learning performance.

The next set of analyses concern the effect of and between-speaker variance. We keep both mechanisms of forgetting, to see if the performance of naive and Ebbinghaus models can be separated in terms of this new factor.

### Between-speaker variance

**Figure 7:**
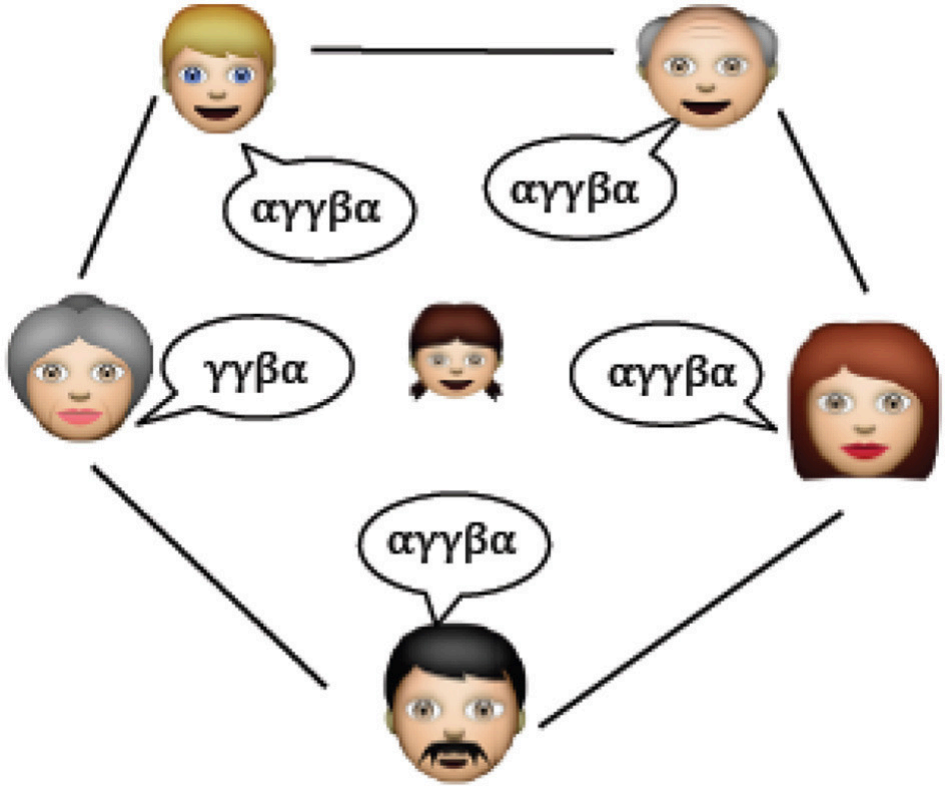
Between-speaker variation. The child faces multiple sources of information when learning her language not all of which are entirely consistent with one another. There is some overlap between speakers, after all, this is partly what defines them as members of the same speech community. But importantly they do not overlap entirely—each person has their own idiolect.

So far we have considered two types of noise. Referential ambiguity and within-speaker variance noise where the word-referent mappings are generally not one-to-one, so each adult has a certain flexibility in the choice of words when talking about a fixed referent. We now introduce a third source of noise that formalizes the notion that different speakers are not guaranteed to use the same linguistic items in exactly the same way, even if they are members of the same speech community (Figure 7) The prevailing wisdom in linguistics has been that adults that talk the same language converge on the same grammar (Crain and Lillo-Martin, 1999, p. 9; Seidenberg, 1997, p. 1600; Nowak et al., 2001, p. 114). Psycholinguistic experiments have begun to question this assumption, showing that significant variation exists in adults' use of a number of canonical grammatical constructions (Brooks and Sekerina, 2006; Dabrowska, 2008, 2012; Street and Dabrowska, 2010). While the case for grammar has proven controversial, the claim that people of the same speech community have different (but overlapping) vocabularies should be less controversial. We therefore implement idiosyncratic language use at the level of lexicon.

**Figure 8:**
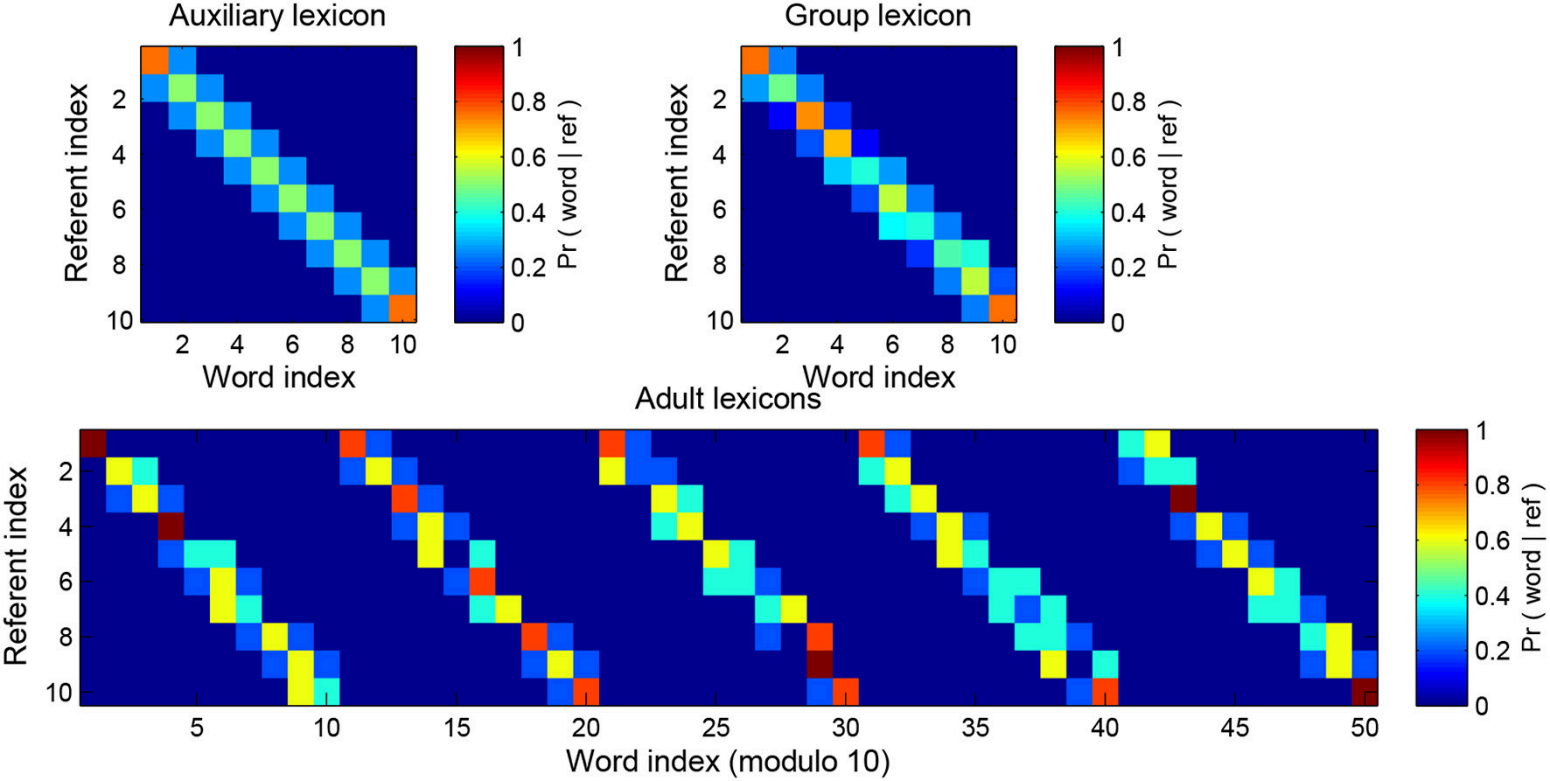
The diversity-generating procedure. The top left matrix shows the auxiliary lexicon from which the 5 adult lexicons shown below have been sampled (it is the tri-diagonal lexicon of Table 1). The more samples that are taken from the original lexicon the closer the adult is to the original lexicon and to the other adults; in this example the number of samples is s = 5. Low sampling equals high between-speaker diversity and high sampling equals low between-speaker diversity. Performance is judged against the aggregate lexicon of all adults (top right). The real world analog here is that the child is effectively judged against the sum total of lexicons it might encounter while learning.

We implement between-speaker variance as follows. We start by building an auxiliary lexicon *F*(*r, w*) which is once again a list of probability distributions over words, one for each referent. We build adult *j*'s distribution *A*^*j*^(*r, w*) by drawing *s* samples from the *F*(*r, w*) distribution. Then as usual we sum the *N*_*A*_ adult lexicons to construct the group lexicon, $G\left(r,w\right)=\;\frac{1}{N_{A}}\overset{N_{A}}{\underset{j=1}{\sum }}A^{j}\left(r,w\right)$. The higher the number of samples *s* used to build the adult lexicons, the higher the similarity of *F* and *G*, and of any pair of adult lexicons *A*^*j*^ and *A*^*k*^, i.e., the lower the diversity in the population of speakers. An example of this process is given in Figure 8.

**Figure 9:**
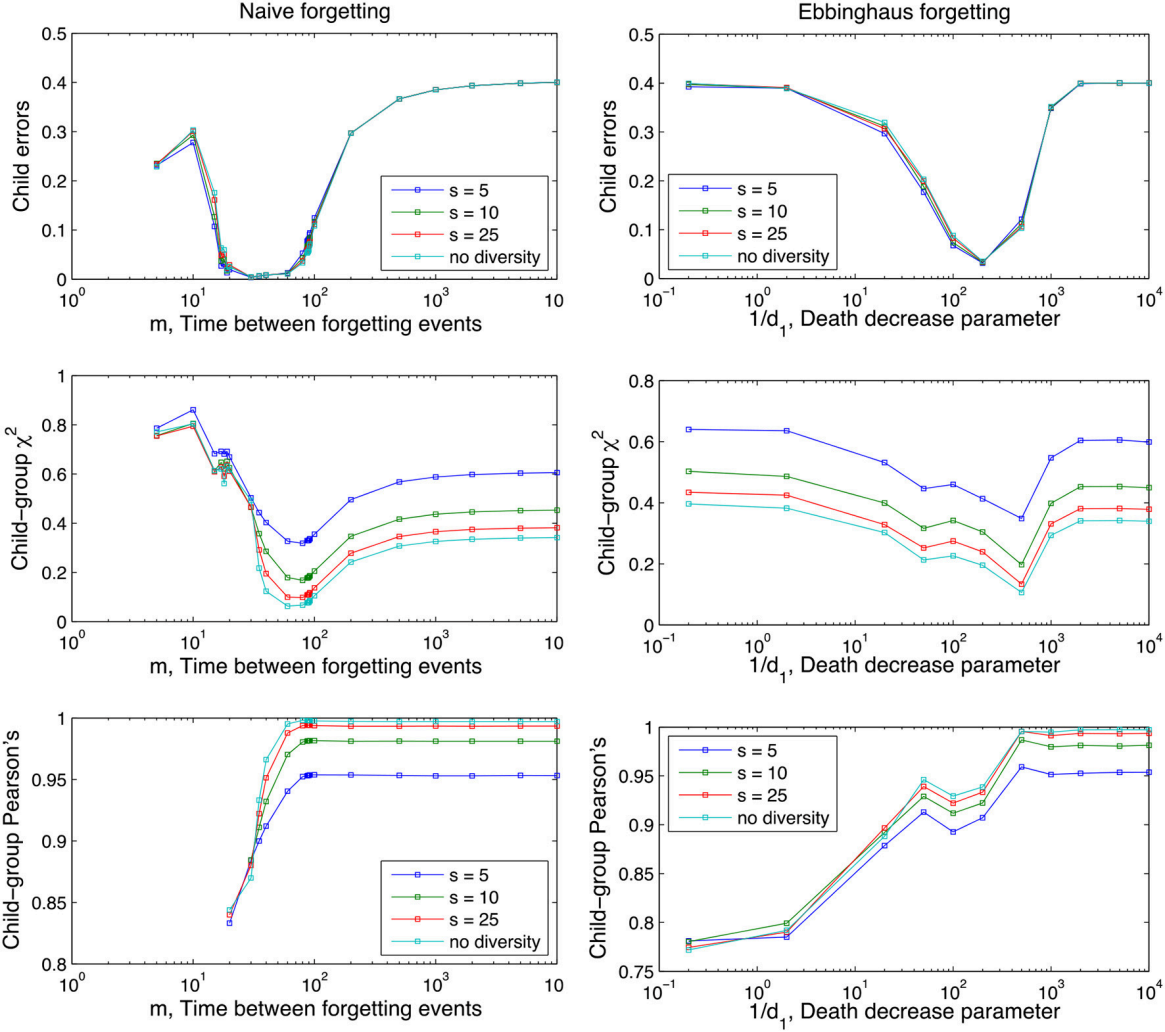
Effect of different degrees of inter-adult diversity on performance as a function of the parameters of the two forgetting mechanisms (*m* and 1/*d*_1_; see Equation 1 and section The Forgetting Mechanisms). Lower *s* values denote higher between-speaker variance. “no diversity” is equivalent to the situation in the previous two analyses of sections The Role of Forgetting and Between-Speaker Variance. A uniform word-use distribution is used. Averages are taken over 100 runs and other parameter values are as in Figures 3, 4.

The sum of all adult's lexicons, the group lexicon, is the object against which the child's progress is measured. As before, performance is judged by our three measures after 10^4^ iterations and as a function of the forgetting mechanism, Figure 9.

As before, reduction in the Child Error scores and a convergence of the child lexicon to the adult lexicons demonstrates the model is learning the intended word-referent pairs, and approximating the associative strength of these pairs that is shared by the community it is learning from. In summary, Figure 9 demonstrates XSL is a robust enough learning mechanism to converge of intended word-referent pairs despite between-speaker variance, a situation the child does find themselves in everyday communicative contexts. As before, both naive and Ebbinghaus mechanisms show storage-loss optima under all values of *s* with performance between values only distinguishable on the Chi-squared and Pearson's measures.

## General discussion

For the language learner, multiple sources of indeterminacy or noise provide a fuzzy and probabilistic relationship between the words people use and the world to which they refer. Clearly children do learn despite this indeterminacy, so at a general level, it is possible to think of language acquisition as a signal detection task that takes place in a noisy environment.

Our computational model learned word-referent pairs under three types of noise: referential ambiguity, within-speaker variance and between speaker variance. The implication is that XSL is powerful enough to be useful to the child born into a world where speakers use words ambiguously, where they use different words for the same referents and where different speakers use different words for the same referent. The model achieved this performance without incorporating any specific cognitive biases of the type proposed in the constraints and principles account (e.g., Markman, 1989, 1992; Golinkoff et al., 1994) and without any prescribed developmental changes in the underlying learning mechanism.

Instead we were interested in the extent to which word learning could benefit from being integrated with the domain-general cognitive capacity of memory (and forgetting). By implementing different regimes of forgetting, we found a U-shaped function of the Child Errors from Experiments 1 and 2 that points to a “Goldilocks” zone of forgetting: an optimum store-loss ratio that is neither too aggressive nor too weak, but just the right amount to produce better learning outcomes.

We suggest that the reason for this is that forgetting disproportionally affects the noise as it has fewer tokens to delete from experience than the signal and will therefore more frequently approach a zero association. In the case of spurious associations this effect directly improves the performance of the model as any non-zero associations that exist in the child that do not exist in the adult are recoded as errors. This adds a mechanistic insight in to the experimental evidence that forgetting can improve word-learning and concept abstraction (forgetting-as-abstraction account; Vlach et al., 2008, 2012; Delaney et al., 2010; Vlach and Sandhofer, 2012; Toppino and Gerbier, 2014; Vlach, 2014). Vlach (2014, p. 165) suggested “forgetting promotes abstraction by supporting memory for relevant features of a category and deterring memory for irrelevant features of a category.” In our model, we suggest that this situation comes about when forgetting acts as a high-pass filter that actively deletes (part of) the referential ambiguity noise.

XSL models are examples of a wider trend in linguistics toward adopting a more probabilistic approach to syntactic and lexical processing and representing language in more dynamic and graded terms (e.g., Harris, 1981; Ellis, 2002; MacDonald and Christiansen, 2002; Jurafsky, 2003; Taylor, 2003). The incremental and probabilistic approach of the model means it never completely stops learning or readjusting the weights of associations—although this weight adjustment is more significant early on in development which is why the learning curves are more erratic at the start. The flexibility in the XSL method allows for life-long language learning and readjustment. Dabrowska has shown significant variation in competence in the adult population on a range of canonical language forms (Dabrowska, 2008, 2012; Street and Dabrowska, 2010). The fact that adult performance on “core grammar” such as passives, complex sentences, quantifiers, and morphological inflection can be significantly boosted after intensive exposure to these forms (i.e., training), shows that any learning system needs to capable of readjustment even late on in development.

The constant revisions of XSL may also be considered one of its weaknesses and is the reason that the errors in our model never reach zero. However, the learning curves do show that learning stabilizes. This is because revisions to associative strengths are divided by the denominator of all previous events. The result is that greater and greater evidence is required later on in development to overturn an association. This process is similar to the idea of entrenchment or canalization whereby a linguistic unit is established as a cognitive routine the more it is “rehearsed” in the mind of the speaker (Langacker, 1987). Entrenchment is a matter of degree and essentially amounts to strengthening whatever response the system makes to the inputs that it receives (Hebb, 1949; Allport, 1985). Once this entrenchment is established as a routine it can be difficult to reverse. For example, Japanese speakers find it difficult to discriminate between /r/ and /l/ because it activates a single representation, whereas for English-speakers the two representations remain separately entrenched (Munakata and McClelland, 2003).

One might argue that due to the probabilistic nature of cross-situational learning means the correct referent should always emerge from the noise, so what is the added value of forgetting? It is true that the correct referent has the highest probability of emerging from the noise if enough time has elapsed. In previous models, that probability is actively increased due to extra reinforcement mechanisms which are usually considered alongside XSL (but which are distinct from XSL) and which serve to accelerate the learning process. For example, Fazly et al. (2010) use an “alignment step” which uses previous evidence to guess meanings (assuming that the child operates in an optimal Bayesian way), so that previous correct evidence is reinforced. Our forgetting mechanism is an alternative way to effectively reinforce the correct pairings by actively reducing the strength of the erroneous pairings (more precisely, of the weaker pairings, which happen to be the erroneous ones due to the nature of XSL). This is in addition to the fact that we need forgetting because (1) people do have imperfect storage, access and retrieval of information (2) some forgetting improves learning performance when compared with models that have no forgetting or too much forgetting.

One conclusion we can draw from this work is that integrating aspects of domain-general cognition into probabilistic/statistical approaches to learning can create more psychologically plausible models and may improve model performance (Elman, 1993; Ibbotson and Tomasello, 2009; Ibbotson et al., 2012, 2013a,b, 2018; Kachergis, 2012; Tilles and Fontanari, 2012; Ibbotson and Kearvell-White, 2015; Yurovsky and Frank, 2015; Kachergis and Yu, 2017).

More generally, the fact that integrating a plausible account of memory improves word learning provides further support for the view that the complexity of language emerges through the interaction of cognition and language use over time (Langacker, 1987, 1991; Croft, 1991; Givón, 1995; Tomasello, 2003; Goldberg, 2006; Bybee, 2010).

The role of forgetting has been argued to have an important role not just in learning associations but generalizing knowledge to new instances—a fundamental part of the creative aspect of acquiring a language (Vlach et al., 2008, 2012; Vlach and Sandhofer, 2012; Vlach, 2014). Following Vlach (2014), we add further support to the idea that the mechanism that researchers have traditionally thought of as inhibiting learning—forgetting—may actually promote learning words. We add to this account that it is not just “forgetting” but the right amount of forgetting and the reason why this amount of forgetting works. Learning is boosted in the Goldilocks zone of forgetting where memory for noisy associations is deleted, intended referents are retained, and the signal is effectively amplified. Vlach (2014, p. 168) “Parents, educators, and scientists may want to reconsider a long-held, intuitive assumption that forgetting uniformly constrains children's ability to learn.”

## Author contributions

PI conceived the original idea, designed the model and wrote the paper. DL implemented the model. AM provided supervision.

### Conflict of interest statement

The authors declare that the research was conducted in the absence of any commercial or financial relationships that could be construed as a potential conflict of interest.
