# Supplementary material for: Goldilocks Forgetting in Cross-Situational Learning
Source: Front Psychol. 2018 Aug 15;9:1301. doi: 10.3389/fpsyg.2018.01301 (PMC6104671; doi:10.3389/fpsyg.2018.01301)
Supplement: Supplementary file 1 [file Data_Sheet_1.docx]

**APPENDIX 1**

The objects shown in Table 1 can be constructed as follows. Let $P_{1}(r_{i})$ be the probability of picking $r_{i}$ as a target referent. Let $P_{2}(r_{k}|r_{i})$ be the probability that referent $r_{k}$ is picked as a distractor given that referent $r_{i}$ has just been picked as a target (this conditioning is needed because target and distractor referents are drawn without replacement, and we are dealing with a small number of referents). Let $P(w_{j}|r_{i})$ stand for the $(i,j)$-th adult lexicon element (probability that the adult will utter word $w_{j}$ when talking about referent $r_{i}$); in our examples so far we have always taken this lexicon matrix to be (tri-)diagonal.

Call $p_{ij}$ the probability per iteration that the child will add one token to her (non-normalized) lexicon element $(r_{i} ,w_{j})$. That can happen if referent $r_{i}$ is picked as a target, but also if it is picked as a referent. The two terms below take care of those two cases respectively:

$$p_{ij} = P_{1}\left( r_{i} \right) P\left( w_{j} \right|r_{i}) + \sum_{k\neq i} P_{1}\left( r_{k} \right) P\left( w_{j} | r_{k} \right) P_{2}\left( r_{i} | r_{k} \right) .$$

The child's lexicon elements are then normalized per referent, so that each line constitutes a probability distribution over words, for a given referent. The normalising factor for row $i$ is therefore

$$q_{i} = \sum_{j} p_{ij} = P_{1}(r_{i}) + \sum_{k\neq i} P_{2}\left( r_{i} | r_{k} \right) P_{1}\left( r_{k} \right) ,$$

i.e. the probability of adding a token anywhere on row $i$ is the sum of the probabilities that referent $i$ will be picked as a target and that it will be picked as a distractor.

We can now name the objects pictured in Figure 10. The top matrix is the adult lexicon $P(w_{j}|r_{i})$. The remaining matrices are

$$m_{ij} = \frac{p_{ij}}{q_{i}} ,$$

for the three different types of target referent distribution $P_{1}(r_{i})$

$$P_{1}^{uniform}(r_{i})=\frac{1}{R}$$

$$P_{1}^{Zipf}\left( r_{i} \right)= c i^{-\gamma}, c=1/\sum_{k=1}^{R} k^{-\gamma}, \gamma=1$$

$P_{1}^{gauss}\left( r_{i} \right)=c^{'}\exp\frac{-\left( i-i_{0} \right)^{2}}{2\sigma^{2}}, c^{'}=1 /\sum_{k=1}^{R} \exp\frac{-\left( k-i_{0} \right)^{2}}{2\sigma^{2}}, i_{0}=0, \sigma=4$ .

(Eq. 6)

The distractor referent distribution $P_{2}(r_{i}|r_{j})$ is identical to the target one $P_{1}(r_{i})$, except for a missing $j$-th term in the normalisation factors that captures the sampling without replacement (of the referent that has been picked as a target).

**APPENDIX 2**

**Examples of the effect of the memory parameter**

The following figures show examples of the time evolution of part of the child’s lexicon (the particular row $c(w | r= 4)$ which represents the associative strength of words $w = 1,2,3,\ldots$ with referent number 4), for different values of the memory parameter $m$, using the naïve forgetting mechanism. They illustrate that when forgetting events are too frequent, not enough information is retained to constitute a signal (first figure, $m=17$), while when forgetting events are too rare, too much erroneous information (originated by the referential ambiguity inherent to the learning context) is retained (third figure, $m=1000$). The best signal is obtained in the intermediate regime where the erasing of errors (via forgetting) best matches the rate at which errors are recorded due to the continuous exposure to distractor referents (second figure, $m=40$). In these examples the goal is to learn the $r=4$ line in the adult’s lexicon of Figure 1 of the main text, where $P(w=3 | r=4) = 0.25$, $P(w=4 | r=4) = 0.5$, $P(w=5) | r=4) = 0.25$ and all other elements are null. Note that for clarity the figures below show the *non-normalized* child lexicon, i.e. just the net number of tokens that support the different word-referent associations considered.

|  |  |
| --- | --- |
|  | |
